# Occult hepatitis B infection: an evolutionary scenario

**DOI:** 10.1186/1743-422X-5-146

**Published:** 2008-12-11

**Authors:** Formijn J van Hemert, Hans L Zaaijer, Ben Berkhout, Vladimir V Lukashov

**Affiliations:** 1 Laboratory of Experimental Virology, Department of Medical Microbiology, Center for Infection and Immunity Amsterdam (CINIMA), Academic Medical Center, University of Amsterdam, Amsterdam, the Netherlands; 2 Laboratory of Clinical Virology, Department of Medical Microbiology, Center for Infection and Immunity Amsterdam (CINIMA), Academic Medical Center, University of Amsterdam, Amsterdam, the Netherlands

## Abstract

**Background:**

Occult or latent hepatitis B virus (HBV) infection is defined as infection with detectable HBV DNA and undetectable surface antigen (HBsAg) in patients' blood. The cause of an overt HBV infection becoming an occult one is unknown. To gain insight into the mechanism of the development of occult infection, we compared the full-length HBV genome from a blood donor carrying an occult infection (d4) with global genotype D genomes.

**Results:**

The phylogenetic analysis of polymerase, core and X protein sequences did not distinguish d4 from other genotype D strains. Yet, d4 surface protein formed the evolutionary outgroup relative to all other genotype D strains. Its evolutionary branch was the only one where accumulation of substitutions suggests positive selection (dN/dS = 1.3787). Many of these substitutiions accumulated specifically in regions encoding the core/surface protein interface, as revealed in a 3D-modeled protein complex. We identified a novel RNA splicing event (deleting nucleotides 2986-202) that abolishes surface protein gene expression without affecting polymerase, core and X-protein related functions. Genotype D strains differ in their ability to perform this 2986-202 splicing. Strains prone to 2986-202 splicing constitute a separate clade in a phylogenetic tree of genotype D HBVs. A single substitution (G173T) that is associated with clade membership alters the local RNA secondary structure and is proposed to affect splicing efficiency at the 202 acceptor site.

**Conclusion:**

We propose an evolutionary scenario for occult HBV infection, in which 2986-202 splicing generates intracellular virus particles devoid of surface protein, which subsequently accumulates mutations due to relaxation of coding constraints. Such viruses are deficient of autonomous propagation and cannot leave the host cell until it is lysed.

## Background

Occult HBV infections are defined as the presence of HBV DNA and the absence of HBV surface antigen (HBsAg encoded by the S gene) in plasma or serum of HBV-infected patients [1]. This infection may persist in individuals for years without emerging symptoms of overt HBV infection. Co-infection [2], drug abuse [3] or immunosuppression [4] can trigger an enhancement of HBV DNA levels without an increase of HBsAg. Transmission of HBV from individuals with occult HBV infection may occur via organ transplantation or blood transfusion [5]. It is presently unclear to what extent occult HBV infection represents a risk factor for the community other than for the infected individual [6].

In HBV sequences obtained from serum samples of HBsAg seronegative carriers, a plethora of mutations has been observed [7-10]. Point mutations, deletions and splicing alternatives have been associated with occult HBV, but it is unclear whether these mutations are a cause or a consequence of an occult HBV infection. Many of these occult infection associated mutations reside in the S gene and/or regions governing the regulation of S gene expression, but they have also been documented for the core (C) and polymerase (P) genes.

Replication-defective mutants of HBV have been detected in the circulation of symptom-free individuals as early as 1987, and a notable example showed a deletion in to the pre-S region [11], which mediates cellular receptor binding [12]. Subsequently, splicing of viral RNA has been identified as a major cause of HBV genome and particle heterogeneity [13-16]. Spliced viral mRNA may become translated into aberrant HBV proteins with unknown function [17]. The existence of a potential splice site does not necessarily mean that it is constitutively used. A region called PRE (Posttranscriptional Regulatory Element) has been identified in the HBV genome. The PRE facilitates the export of PRE-containing transcripts from the nucleus to the cytoplasm [18-20]. Consequently, viral transcripts reach the cellular translational machinery along two competing pathways: either being promoted by PRE before splicing occurs or via the regular export route of spliced cellular mRNAs. More recently, Hass and coworkers referred to this competitive feature to demonstrate that integrity of the 458/459 exon/intron transition is required for the accumulation of pre-S2/S mRNA ([21] see also *editorial*). Posttranscriptional reduction of surface protein and mRNA expression to a background level was due to a single G458A substitution [21] and could also be caused by deletion of 30 nucleotides immediately downstream of this site [22].

Recently, we obtained sequence information for HBV strains present in occult infections [7]. Based on its analysis, we here propose a novel splicing event of HBV RNA (deleting the nucleotides from 2986 to 202) that abolishes surface protein expression without affecting other functions encoded in the virus genome (P, C and X). HBV strains prone to this splicing opportunity constitute a separate clade in a phylogenetic tree of the genotype D polymerase sequences. In this clade, a T-to-G mutation at position 173 truncates a splice-promoting polypyrimidine tract [23] and also affects the local secondary structure of the viral RNA [24]. As a result, the splicing activity at the neighboring 202 splice acceptor site may be down-regulated. The splicing possibility (2986-202) based on NetGene2 predictions presently awaits further experimental support by analysis of liver samples, which are much more complicated to obtain from healthy occult HBV carriers than blood samples.

## Results

### Mutations in occult EU155893 HBV DNA

**Figure 1:**
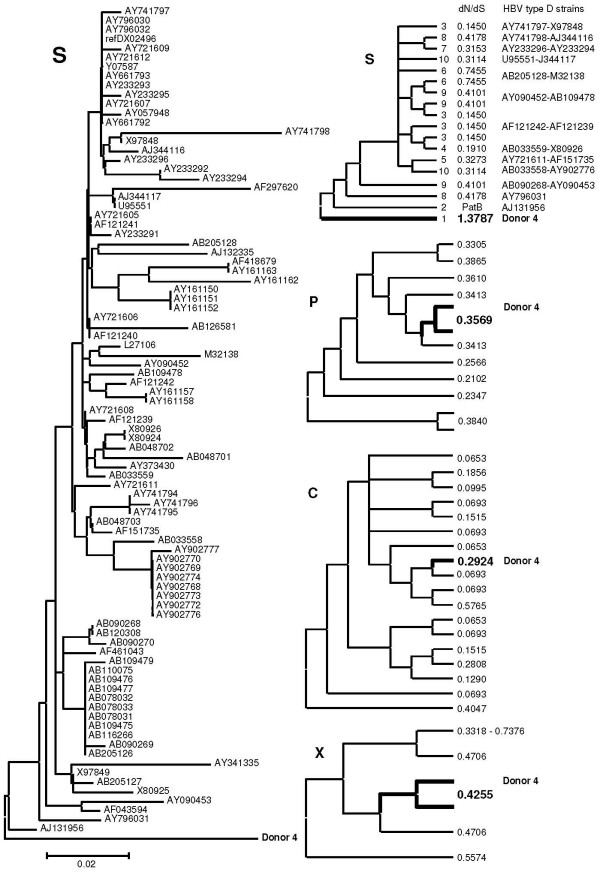
**HBV strain phylogeny**. A bootstrap consensus tree based on JTT-estimates of amino acid replacements in surface proteins of HBV genotype D displays the surface protein of donor 4 carrying an occult infection in the outgroup position (left panel). The scale bar indicates 2% of evolutionary divergence. For phylogenetic analysis by maximum likelihood, the HBV type D strains were grouped according to their topological position, approximately and provided with labels as indicated next to the branches of the compressed topology tree (right panel, S). The corresponding values obtained for dN/dS are in between of the labels and strains columns; PatB means ''parameter at boundary''. Data on donor 4 are in bold-face. The three panels marked by P(olymerase), C(ore) and X were constructed in a similar fashion, but without mentioning GenBank IDs and clade/branch labels. In case of P and X, the donor 4 species was combined with its nearest neighbor in order to avoid deviation due to insufficient branch length.

HBV surface protein of donor 4 with an occult HBV infection (EU155893, d4) takes the outgroup position in a bootstrapped phylogenetic tree based on JTT-estimates of amino acid replacements in genotype D surface proteins (Fig 1, left panel). The lengths of the branches of the available surface protein sequences from the other donors with occult HBV infection (1a, 1b, 2, 3, 5a and 5b) were similar or even larger than the d4 branch length leading to severe tree compression and were therefore excluded from the tree. PAML analysis allowing dN/dS values of clades and branches to exceed the value of 1 generated a dN/dS value of 1.3787 for the branch of d4 surface protein gene, almost a fourfold of the average value of 0.3579 ± 0.1831 (range 0.1450–0.7455) of the other clades and branches (Fig 1, right panel, S). A likelihood ratio comparison with a similar analysis limiting dN/dS values to maximally 1 provided statistical support (p < 0.001). In the other HBV genes, the dN/dS values of d4 DNA were close to the average values (Fig 1, right panel, P, C and X) – P: 0.3162 ± 0.0656 (range 0.2102–0.3840), C: 0.2180 ± 0.1733 (range 0.0653–0.5765) and X: 0.5136 ± 0.1490 (range 0.3318–0.7376). These data indicate the presence of positive selection or relaxed selective constraints as a characteristic property of the surface protein gene in this case of occult infection. During evolution from an overt to the present occult infection, the surface protein gene of d4 HBV accumulated non-synonymous and synonymous nucleotide substitutions to approximately equal proportions.

**Figure 2:**
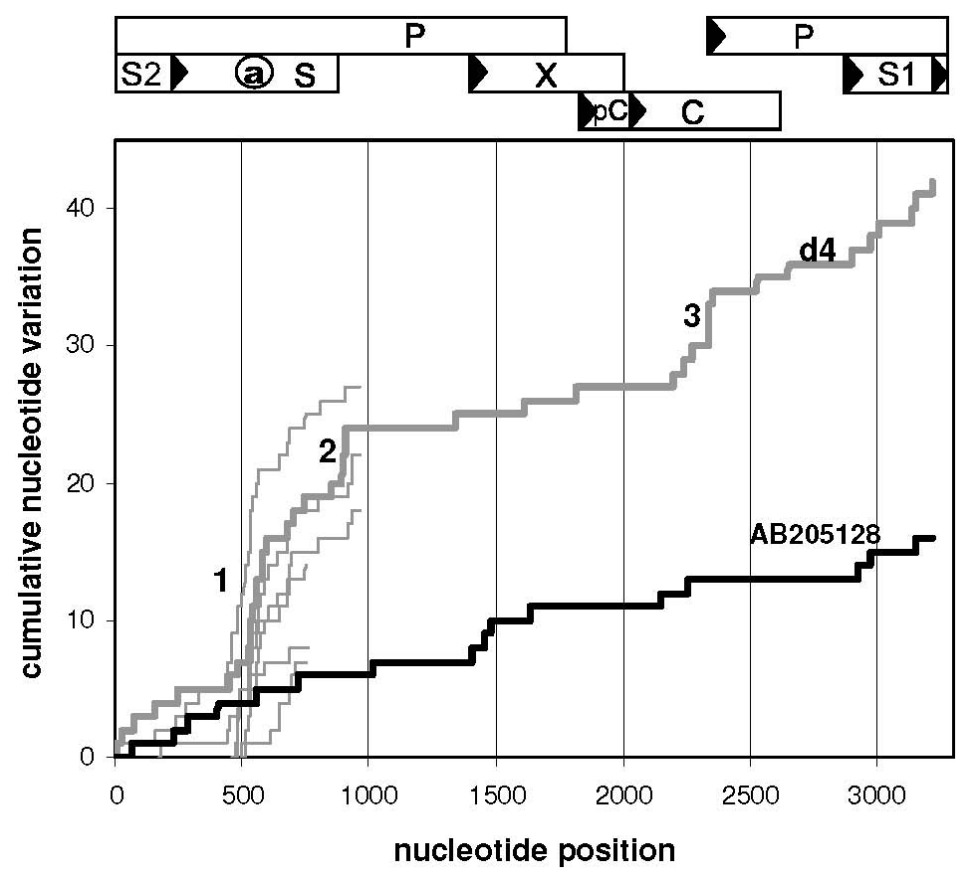
**Mutational scan along the HBV genome**. Nucleotide substitutions uniquely present in EU155893 HBV DNA (d4, thick grey line, occult infection) and in control AB205128 HBV DNA (thick black line, overt infection) are compared with 89 HBV DNAs of genotype D and plotted cumulatively along the HBV genome. Steep slopes at the a-determinant (1), the oligonucleotide 895–909 (2) and the central part of C (3) indicate the relatively high divergence of these regions in d4 HBV. Thin grey lines represent characteristic mutations in the available HBV sequences from blood samples of the other donors with occult HBV infection. Numbering starts from the conventional EcoR1 site between S1 and S2. A map of HBV genome organization is provided on top of the figure.

The HBV genome of d4 contains 42 unique nucleotide substitutions that are not observed in a collection of 89 genotype D HBV species (DQ series [8] were not included, see below). In control strain AB205128 from a patient with overt HBV infection, only 16 characteristic mutations had accumulated in the genome. In order to pinpoint clusters of d4-specific substitution, we awarded each of these mutations a value of 1 and plotted the mutational hits cumulatively along the genome (Fig 2). Steep increases of the plot indicate regions of enhanced divergence, which is prominent in d4 HBV DNA at the a-determinant region (10/42 substitutions), the oligonucleotide 895–909 (4/42) and the central part of the core protein (5/42). As far as sequences are available, accumulation of nucleotide substitutions specifically at the a-determinant region is also prominent in strains from other donors with occult HBV infection (Fig 2, thin lines: 1a, 1b, 2, 3, 5a and 5b). Conservation prevails in X protein, the N-terminal part of S and in the remaining parts of core and polymerase. S1, S2, and C-terminal parts of S display an intermediate degree of variation. In the control strain AB205128, local accumulation of mutations can hardly be observed and slopes are similar to those of HBV d4 DNA in the conserved regions. Enhanced mutational rates at sites are usually associated with a relaxation of functional constraints of the regions involved and may indicate a contribution of these regions to the evolutionary transition from an overt into an occult HBV infection. A diminished interaction between core and surface proteins due to the mutations introduced at the regions 1 and 3 of HBV d4 DNA (Fig 2) may provide a substantiation of this process, rendering the transition irreversible.

**Figure 3:**
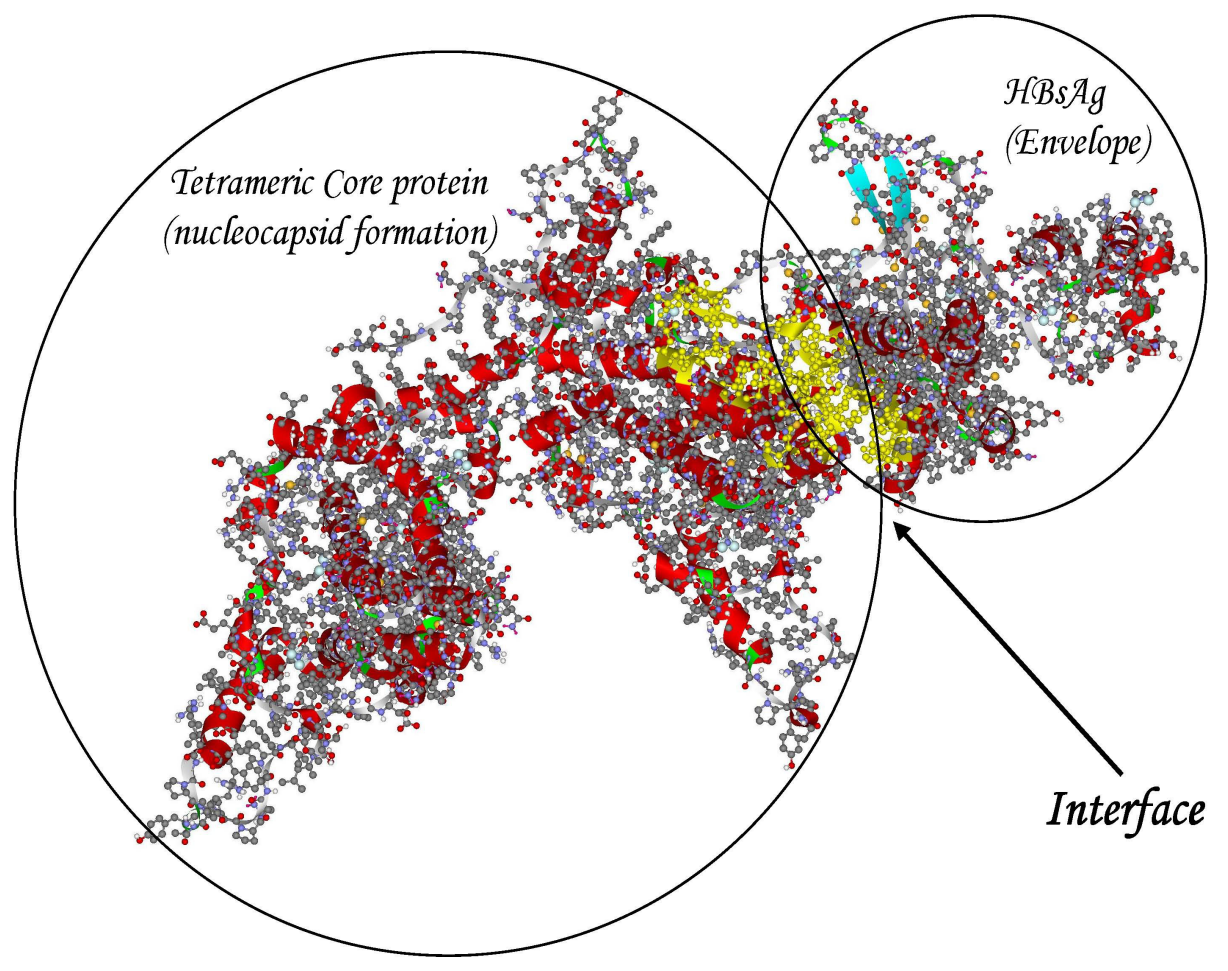
**Model of the core/surface protein interaction**. A 3D-modeled complex of tetrameric core protein with HBsAg monomer shows the yellow-colored amino acid residues comprising the interface between the two proteins.

We have previously studied the amino acid composition of interfaces between 3D-structured domains or proteins of HBV [25] by means of computational alanine replacement scanning [26]. The docking procedure [27] of monomeric HBsAg with tetrameric core protein (PBD entry 1qgt) followed by ALASCAN-directed selection among the alternative structures resulted in the complex with a yellow-colored interface region as shown in Fig 3. A PDB formatted data file carrying the coordinates of the complex is provided online as Additional File 1. The corresponding output of the ALASCAN server shows that the central part of core protein (amino acid residues 67–96), the N-terminal half of the a-determinant region (96–122) and the C-terminal part of surface protein (169–195) participate in the interface between core and surface proteins (Table 1) in order to promote the formation of an infectious virus particle. In d4 DNA, these regions display the d4-characteristic feature of enhanced sequence divergence. Not all of these nucleotide substitutions translate into amino acid replacements. Replacements typical for d4 HBV are G74V, I80A and Y100C in core and P111S, T123P, T125I, L175S and M197T in surface protein, respectively. These results indicate the evolutionary loss of the ability for S/C interface formation during the development from a "wild type" genotype D ancestor to the occult d4 phenotype. It should be kept in mind that gene overlapping constraints does not preclude the independent evolution of genes in HBV [28].

**Table 1:** Core/Surface protein interface in HBV refD_X02496.

	**Core protein**				**Surface protein**			
	Pos	AAres	ΔΔG	Pos	AAres	ΔΔG		
C	67	Thr	0.15	96	Val	0.35	left @	
C	70	Thr	0.49	100	Tyr	1.03	left @	
C	71	Trp	0.69	103	Met	0.48	left @	
C	74	Gly	0.75	104	Leu	0.45	left @	
C	75	Asn	-0.24	113	Ser	0.31	left @	
C	76	Leu	-0.05	114	Ser	0.52	left @	
C	77	Glu	1.83	116	Thr	0.04	left @	
C	78	Asp	1.05	117	Ser	0.98	left @	
C	82	Arg	7.02	118	Thr	-0.02	left @	
C	83	Asp	6.39	121	Cys	0	left @	
C	84	Leu	0.13	122	Arg	0.55	left @	
C	86	Val	0.15	169	Arg	1.26	S	
C	88	Tyr	0.06	173	Leu	0.49	S	
C	91	Thr	0.13	174	Ser	1.69	S	
C	92	Asn	0.61	175	Leu	0.72	S	
C	95	Leu	0.56	177	Val	0.32	S	
C	96	Lys	-0.02	181	Gln	0.11	S	
					
				182	Trp	0.26	S	
				191	Trp	0.29	S	
				192	Leu	0.14	S	
				195	Ile	0.27		

ΔΔG indicates the amount of energy, by which the complex becomes destabilized by the computational replacement of the corresponding residue (AAres) with alanine.

Note: Labels C, left @ and S correspond to map positions in Fig 1.

### Altered RNA splicing in occult d4 HBV

**Figure 4:**
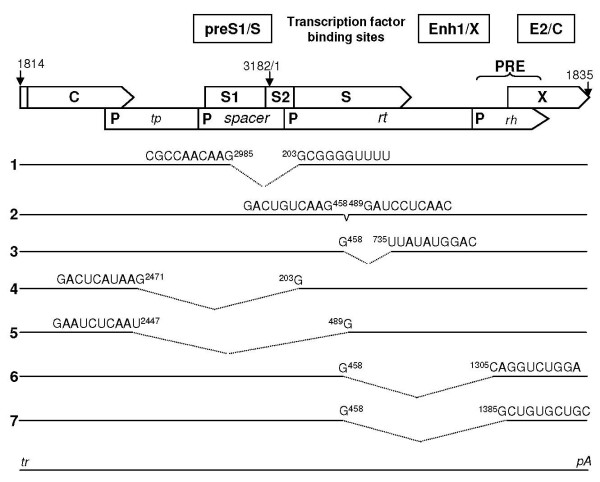
**RNA splicing possibilities in the HBV genome**. Splice patterns in HBV pregenomic RNA are predicted by NetGene2. Genome organization is presented as functional domains in pgRNA decorated with transcription factor binding sites (upper two panels) from terminal repeat (tr) to polyadenylation sequence (pA) (bottom line). PRE indicates the position of the Posttranscriptional Regulatory Element. Different modes of splicing are numbered and referred to in the text.

Splicing of HBV RNA is considered not to be essential for HBV propagation. Intriguingly, an association was reported between RNA splicing and the generation of replication-defective HBV variants [13-17]. We applied the NetGene2 prediction server in search of characteristic differences between the patterns of donor and acceptor splice sites in the HBV genomes of d4 and X02496 as genotype D reference strain (Table 2). In many aspects (position, phase and confidence), the splicing possibilities are quite similar for these strains, except for the presence of an extra acceptor site at position 202 in the DNA of d4 HBV. Interestingly, a splicing event between the acceptor site 202 and the donor splice site at position 2986 preserves the original reading frame, but deletes almost the entire spacer region from the viral polymerase and – in the overlapping S gene – the S-promoter region and the 5'-untranslated leader together with 16 N-terminal codons of preS2/S mRNA (Fig 4, case 1). Consequently, the polymerase-dependent functions in virus replication (terminal protein – *tp*, reverse transcriptase – *rt *and RNAse H – *rh*) remain unaffected, while sequences for large, middle and small surface protein gene expression in the overlapping reading frame are deleted. As a result of this post-transcriptional event, a virus genome may regularly replicate and be encapsidated inside the host cell, but cannot be enveloped and hence has lost the ability to exit the host cell and to enter new cells. These molecular properties match the characteristics of occult HBV infection.

**Table 2:** Splice sites in donor 4 HBV as predicted by NetGene2.

Donor splice sites					
	pos 5'->3'	phase	strand	confidence	5' exon intron 3'
1	459	1	+	0.83	GACTGTCAAG^GTATGTTGCC
2	2448	0	+	0.31	GAATCTCAAT^GTTAGTATTC
3	2472	0	+	0.75	GACTCATAAG^GTGGGGAACT
4	2986	0	+	0.99	CGCCAACAAG^GTAGGAGCTG
					
Acceptor splice sites					

	pos 5'->3'	phase	strand	confidence	5' intron exon 3'
**1**	**202**	**1**	**+**	**0.57**	**CGTGTTACAG^GCGGGGTTTT**
2	488	2	+	0.93	CTAATTTCAG^GATCCTCAAC
3	504	0	+	0.07	CAACCACCAG^CACGGGACCC
4	707	2	+	0.17	TGGTTCGCAG^GGCTTTCCCC
5	734	2	+	0.26	TGGCTTTCAG^TTATATGGAC
6	1304	2	+	0.25	TTGCTCGCAG^CAGGTCTGGA
7	1307	2	+	0.28	CTCGCAGCAG^GTCTGGAGCA
8	1384	1	+	0.26	TGGCTGCTAG^GCTGTGCTGC
9	1979	1	+	0.82	TTTCCTTCAG^TACGAGATCT
10	1985	1	+	0.14	TCAGTACGAG^ATCTTCTAGA
11	2335	2	+	0.18	ACTTCCGGAG^GTTGCTGTTG
12	2349	1	+	0.31	CTGTTGTTAG^AGGACGAGGC
13	2351	0	+	0.19	GTTGTTAGAG^GACGAGGCAG
14	2357	0	+	0.19	AGAGGACGAG^GCAGGTCCCC
15	2361	1	+	0.19	GACGAGGCAG^GTCCCCTAGA
16	2370	1	+	0.19	GGTCCCCTAG^AAGAAGAACT
17	2373	1	+	0.19	CCCCTAGAAG^AAGAACTCCC
18	2376	1	+	0.19	CTAGAAGAAG^AACTCCCTCG
19	2394	1	+	0.37	CGCCTCGCAG^ACGAAGGTCT
20	2838	0	+	0.07	TGGGAACAAG^ATCTACAGCA
21	2846	2	+	0.18	AGATCTACAG^CATGGGGCAG
22	2856	0	+	0.19	CATGGGGCAG^AATCTATCCA
23	2870	2	+	0.19	TATCCACCAG^CAATCCTCTG
24	2901	0	+	0.18	CGACCACCAG^TTGGATCCAG
25	2911	1	+	0.07	TTGGATCCAG^CCTTCAGAGC

The 202 acceptor site is mentioned in bold.

Notably, the splice 2986 to 202 is rather unique in this virus-inactivation aspect. Other splice opportunities may not occur due to proximity (459 to 488), may induce a frame-shift (2986 to 488 or 734) or may affect essential viral functions (459, 2472 or 2986 to 707–1384). As shown by zu Putlitz and coworkers [22], deletion of nucleotides 459–488 (Fig 4, case 2) caused a >99% reduction in the level of preS2/S mRNA without affecting the transcriptional rate of this mRNA and the replication competence of the mutant HBV. It may be expected that every splicing event that induces this deletion (Fig 4, cases 3, 5, 6 and 7) similarly affects surface protein expression. Also, it should be noted that the deletion spans the amino acid residues 102–111 in the surface protein frame. This region constitutes the N-terminal domain of the a-determinant and participates in the interface between core and surface protein region (previous section, Table 1). Splicing between 459 and 734 (Fig 4, case 3) also preserves the original reading frame, but the intron/exon boundary resides just at the YMDD motif of polymerase yielding an inactive polymerase. Similarly, splicing between 2472 and 202 (Fig 4, case 4) retains the reading frame, but abolishes – in addition to the spacer region – a majority of the *tp *domain of polymerase.

Splice prediction in human mRNA by means of NetGene2 is a joint assignment method combining consensus sequence information with parameters of coding/non-coding transitions. It could be argued that an overlapping gene structure may interfere with these criteria. However, NetGene2 performs reliably in the prediction of splicing events that have been described to occur (Fig 4). For instance, Hass and coworkers [21] observed that a single G458A mutation prevented splicing from 459 to 1304 or 1384 (Fig. 4, cases 6 and 7). The donor sites 2088, 2448, 2472 and acceptor sites 2351, 2901, 283, 488 have also been identified as contributing to the splicing of HBV RNAs (i.e. Fig 4, case 5), some in genotypes other than D [13-17].

### RNA splicing predictions for HBV genotype D representatives

**Figure 5:**
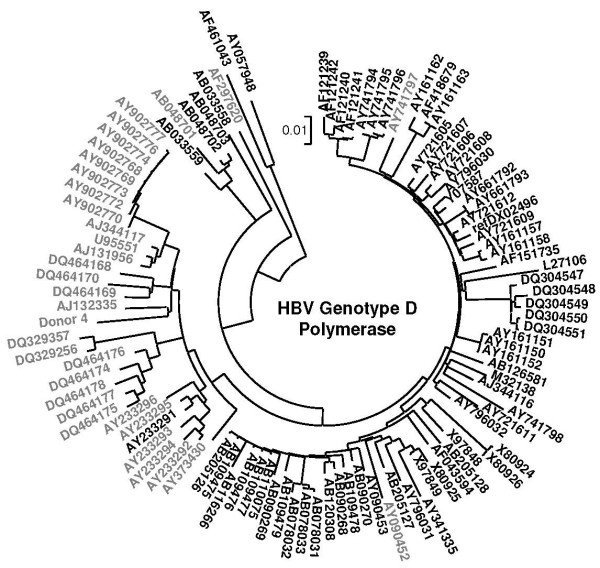
**Detailed phylogeny of HBV genotype D strains**. A phylogenetic bootstrapped consensus tree of HBV genotype D strains was derived from replacements in the amino acid sequences of the viral polymerase. Grey clade members scored positively with respect to the 202 acceptor site predicted by NetGene2, in contrast with members of the black collection. The scale bar indicates 1% of evolutionary divergence.

**Figure 6:**
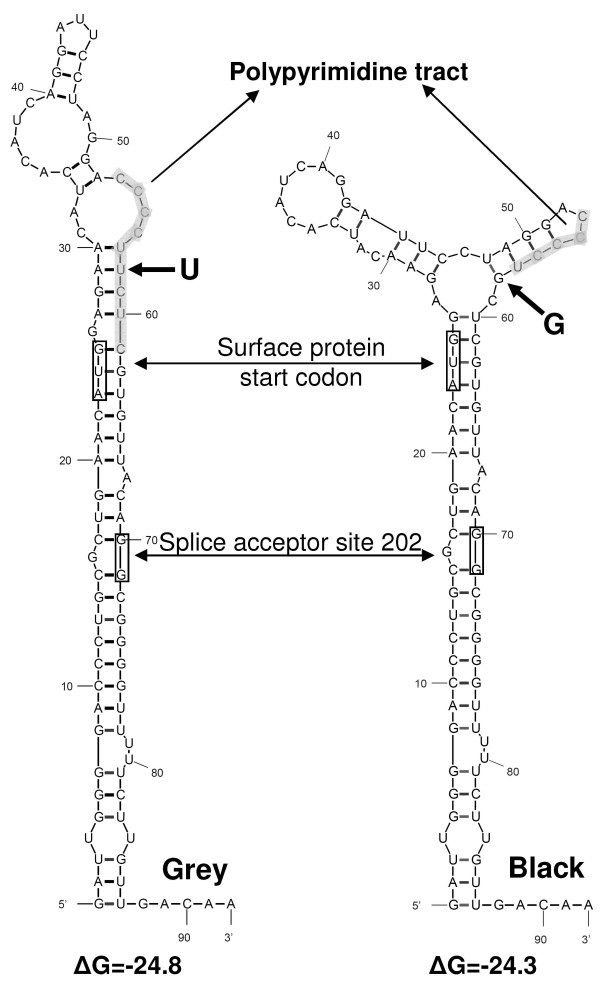
**Analysis of splice acceptor site 202 in the HBV genome**. A single U173G mutation affects the local RNA secondary structure. A consensus sequence of grey clade members (left panel) differs from the black collection (right panel) by an U-versus a G-nucleotide promoting exposure into a loop structure or burial into a stem structure, respectively, of a polypyrimidine tract (marked by shading) obligatory for efficient splicing at the 202 acceptor site indicated by an arrow. For the purpose of orientation, the AUG initiation codon for surface protein translation is also indicated. Values for ΔG are in kcal/mole.

The ability to promote 2986-202 RNA splicing may not be a special property of d4 HBV. In a collection of 104 HBV genotype D representatives, NetGene2 reported another 32 cases. Remarkably, 29 of these strains constitute a separate clade in a phylogenetic tree based on amino acid replacements in the polymerase protein of these viruses (Fig 5). A tree based on amino acid replacements in the large surface protein (not shown) generated a similar result with (A/D recombinant) strain AF297620 at the core of the clade as a neighbor of d4. Genotype D representatives outside this clade (referred to as the "black collection") may differ from the true clade members ("grey clade") by a diminished tendency to develop the occult phenotype by means of 2986-202 RNA splicing as marked by clade member d4 HBV. The consensus sequences of the 2986 donor and 202 acceptor sites are present almost ubiquitously among the entire collection and hence, the enhanced scores of the proposed intron sequences may be a distinctive property of the clade members. To explore the proposed intron sequence in more detail, we compared a consensus polymerase sequence from the "grey" clade with that of the "black" collection and found 7 nucleotide differences between the proposed intron regions of these two sequences. Solely, the 7^th ^mutation T173G displayed the ability of changing a grey phenotype (T) into a black one (G) and vice versa. This mutation is synonymous in the reading frame for surface protein (^13^Leu) and replaces a Ser (T) for an Ala (G) at the polymerase frame. The nucleotides A (Thr) and C (Pro) have not been found in this position. The T-to-G mutation interrupts a polypyrimidine tract that is likely to promote RNA splicing at the neighboring 202 splice acceptor site [23,29]. Also, the mutation appeared to change the local secondary structure of the RNA (Fig 6). The polypyrimidine tract required for appropriate splicing at the 202 acceptor site is either exposed in a loop structure (grey clade) or buried in a base-paired stem (black collection). It has been reported that changes in local RNA structure can modulate the splicing efficiency [24].

In conclusion, a single nucleotide substitution brings on a bipartition among the genotype D HBVs causing a difference in tendency for 2986-202 RNA splicing and hence for the development of an overt into an occult HBV infection.

## Discussion

We describe a thusfar unrevealed RNA splicing alternative (2986-202) that is prominent in a subset of genotype D HBV strains. Splicing of HBV RNA according to this scenario will suppress the expression of surface proteins and spares the functions dedicated to the core and X proteins and to the functional domains (terminal protein, reverse transcriptase and RNAse H) of the viral polymerase. Consequently, virus genomes do replicate and are being encapsidated properly, but the virions are defective due to the absence of surface protein. These virus particles remain captured intracellularly and their propagation becomes dependent on liver cell division. Their release (without immune-reactive surface protein) to an individual's circulation and immune system depends on the turn-over of the infected liver cells. These properties are typical for HBV variants in blood samples of individuals with occult infection [1] like the HBV strains from the donors 1–5 [7]. Moreover, we observed enhanced accumulation of mutations in the d4 variant compared to "wild-type" genotype D, specifically in regions supposed to be involved in the process of S/C interface formation that is amino acid residues in the a-determinant and the C-terminal part of surface protein and in the central part of core protein. Increased rates of mutation and locally diminished protein functionality correlate with the long lasting period since the d4 individual has cleared an overt HBV infection [7].

Experimental evidence for a causal connection of 2986-202 RNA splicing with occult HBV infection is currently lacking, which is mainly due to the fact that collecting liver biopsies from healthy volunteers with occult HBV infection is much more complicated than obtaining blood samples. When analyzing occult HBV in blood samples, selection is inevitably in favor of HBV variants that have reached the patient's circulation. The results of splice prediction in HBV of frozen liver specimens (DQ series, [8]) indicate that a relation of 2986-202 RNA splicing with occult HBV infection is not based solely on the analysis of HBV in blood samples. Also, HBV variants without cell-leaving capabilities may gradually induce symptoms of chronic hepatitis and cirrhosis as long as HBV gene expression remains detectable [30]. Co-infection [2], drug abuse [3] or immunosuppression [4] may cause the appearance of HBV DNA in blood without detectable HBsAg, due to enhanced turn-over rate of liver cells.

Our observation that genotype D variants susceptible to 2986-202 splicing constitute a clade in the phylogenetic tree derived from 3/4 of the complete genome sequence indicates that minor sequence variations may affect regulators of splicing events in individual HBV strains. We show that a single nucleotide mutation is able to activate *in cis *a previously inactive splice acceptor site. mRNA splicing is a classic example of virus-host interaction and thereby depends on the condition of the infected cell, which worsens by cirrhosis, necrosis or apoptosis. On the other hand, many splicing events interfere with virus genome replication by the deletion of vital polymerase protein domains and/or by a shift in the original reading frame at the donor/acceptor junction. Splicing of the sequence 2986-202 is rather unique in that the viral reading frames as well as essential polymerase functions remain unaffected. The PRE sequence, which overlaps with sequences encoding the RNAse H domain of polymerase, is too far downstream to interfere with the splicing event and remains available for transport of the spliced transcript. From the evolutionary point of view, the purifying selective pressure, which intracellularly guards viral genome replication and its encapsidation to prevent degradation, operates properly in the absence of surface proteins. Amino acid sites prone to relaxation of selective constraints tend to display an enhanced rate of replacement as observed for surface protein in the case of an occult HBV infection, particularly in the a-determinant region overlapping the polymerase sequence, which is absent in reverse transcriptases of other viruses (i.e. avian HBV, [25]). The C-gene region involved in the formation of the core/surface protein interface is not protected by the extra constraints of an overlapping reading frame. In conclusion, there is no selective pressure preventing the formation and intracellular accumulation of encapsidated HBV particles. Hence, the splicing event 2986-202 generates infectivity-deficient virus particles with a life-span as long as that of the infected host cell.

May some of these surface protein deficient HBV variants reacquire the ability to initiate productive infection after a prolonged period of occult infection? Relevant scenarios must include a restoration of virus functionality damaged during the period of latency. From the evolutionary point of view, it is beyond expectation that these deficient viruses are able to achieve this solely by means of random mutation and natural selection within the duration of an individual's life, particularly because virus propagation approaches the zero level. Other options of the virus to regain infectivity and propagation are complementation and/or recombination catalyzed by superinfection of the host cell with another HBV strain. Also, it is likely that a single individual with occult HBV infection may carry quasi-species with different causes of latency waiting for superinfection or other triggers to become reactivated. This scenario gains improbability with time as inactivating mutations will accumulate in the surface protein genes. Finally, a small fraction of the liver cells may escape the scenario towards occult infection and may still continue to produce small amounts of infectious virions, which are effectively scavenged by the immune system of an alert host. These cells may induce a reactivation towards overt HBV infection under conditions of immunosuppression. The duration of occult HBV infection – in particular the impact of accumulated mutations – might be an important parameter in order to discern a superinfection from a reactivated existing HBV infection.

## Conclusion

A novel splicing opportunity of HBV mRNA prevents surface protein expression in HBV genotype D without affecting other gene functions (polymerase, capsid and X-protein). This splicing event may become dominant by intracellular evolution and selection. In this case, S-antigen is no longer produced and E-antigen is still secreted. A minute amount of HBV DNA can be detected in the patient's blood due to regular turn-over of infected cells. These criteria match the definition of an occult HBV infection.

## Methods

Recently, we obtained HBV sequences from five donors with occult HBV infection (donors 1–5, GenBank accession numbers EU155889–EU155895), including a full-length genome (EU155893, d4) and six shorter sequences [7]. All of them belong to genotype D as shown by STAR [31] and NCBI [32] analyses. We compared d4 HBV DNA with other human HBV genotype D full-length genomes that were annotated previously [28]. X02496 was used as a reference sequence for HBV genotype D [33]. HBV numbering starts conventionally at the EcoR1 restriction site. ClustalW [34] was used for alignment purposes. Neighbor-joining trees (500 bootstrap replicates) were built in MEGA3.1 [35] applying pairwise deletion and JTT [36] or Poisson-corrected models of amino acid replacement. Phylogenetic analysis by maximum likelihood (PAML 3.15, [37]) was employed to investigate adaptive evolution in d4 branches among the other genotype D branches in S, P, C and X trees. The free-ratios model 1 of PAML, assuming an independent dN/dS ratio (non-synonymous/synonymous nucleotide substitutions) for each branch, turned out to be too parameter-rich. Therefore, clade and branch labels were introduced in newick-formatted trees and upon analysis by means of model 2, dN/dS ratios of clades and branches were presented as branch labels in compressed versions of topology trees. Procedures on the generation of 3D-structures of proteins [38,39] and the application of computational alanine replacement scanning [26] in order to elucidate the interface composition between surface and core proteins have been described previously [25]. The PDB entry 1qgt [40] was the source of the crystal structure of HBV core protein [41]. Docking two protein structures into a single 3D-complex was attained by applying ClusPro [27]. Prediction of RNA splicing was performed by means of the NetGene2 server [42,43]. Cutoff values for confidence were 50% and 20% for the "nearly all true" qualification of donor and acceptor sites, respectively. BioEdit [44] was used for the construction of consensus sequences. RNA secondary structure predictions were obtained by means of the Mfold algorithm [45].

## Abbreviations

PDB: Protein Data Base (of X-ray structures); ALASCAN: (server performing) computational alanine replacement scanning.

## Competing interests

The authors declare that they have no competing interests.

## Authors' contributions

FJvH performed the analyses, HLZ provided materials, BB and VVL were supervisors and all authors were involved in writing the manuscript.

## Supplementary Material

Additional File 1 **A PDB formatted data file carrying the coordinates of the complex between core and surface protein of HBV genotype D.** Click here for file

## References

[B1] Raimondo G, Pollicino T, Cacciola I, Squadrito G (2007). Occult hepatitis B virus infection. J Hepatol.

[B2] Jeantet D, Chemin I, Mandrand B, Tran A, Zoulim F, Merle P, Trepo C, Kay A (2004). Cloning and expression of surface antigens from occult chronic hepatitis B virus infections and their recognition by commercial detection assays. J Med Virol.

[B3] Garfein RS, Bower WA, Loney CM, Hutin YJ, Xia GL, Jawanda J, Groom AV, Nainan OV, Murphy JS, Bell BP (2004). Factors associated with fulminant liver failure during an outbreak among injection drug users with acute hepatitis B. Hepatology.

[B4] Petzold DR, Tautz B, Wolf F, Drescher J (1999). Infection chains and evolution rates of hepatitis B virus in cardiac transplant recipients infected nosocomially. J Med Virol.

[B5] Gerlich WH, Wagner FF, Chudy M, Harritshoj LH, Lattermann A, Wienzek S, Glebe D, Saniewski M, Schüttler CG, Wend UC (2007). HBsAg Non-Reactive HBV Infection in Blood Donors: Transmission and Pathogenicity. J Med Virol.

[B6] Koppelman MH, Zaaijer HL (2004). Diversity and origin of hepatitis B virus in Dutch blood donors. J Med Virol.

[B7] Zaaijer HL, Torres P, Ontanon A, Ponte LG, Koppelman MH, Lelie PN, van Hemert FJ, Boot HJ (2008). Multiple surface antigen mutations in five blood donors with occult hepatitis B virus infection. J Med Virol.

[B8] Pollicino T, Raffa G, Costantino L, Lisa A, Campello C, Squadrito G, Levrero M, Raimondo G (2007). Molecular and functional analysis of occult hepatitis B virus isolates from patients with hepatocellular carcinoma. Hepatology.

[B9] Chaudhuri V, Tayal R, Nayak B, Acharya SK, Panda SK (2004). Occult hepatitis B virus infection in chronic liver disease: full-length genome and analysis of mutant surface promoter. Gastroenterology.

[B10] Cabrerizo M, Bartolome J, Caramelo C, Barril G, Carreno V (2000). Molecular analysis of hepatitis B virus DNA in serum and peripheral blood mononuclear cells from hepatitis B surface antigen-negative cases. Hepatology.

[B11] Okamoto H, Tsuda F, Mayumi M (1987). Defective mutants of hepatitis B virus in the circulation of symptom-free carriers. Jpn J Exp Med.

[B12] Engelke M, Mills K, Seitz S, Simon P, Gripon P, Schnolzer M, Urban S (2006). Characterization of a hepatitis B and hepatitis delta virus receptor binding site. Hepatology.

[B13] Gunther S, Sommer G, Iwanska A, Will H (1997). Heterogeneity and common features of defective hepatitis B virus genomes derived from spliced pregenomic RNA. Virology.

[B14] Soussan P, Garreau F, Zylberberg H, Ferray C, Brechot C, Kremsdorf D (2000). In vivo expression of a new hepatitis B virus protein encoded by a spliced RNA. J Clin Invest.

[B15] Terre S, Petit MA, Brechot C (1991). Defective hepatitis B virus particles are generated by packaging and reverse transcription of spliced viral RNAs in vivo. J Virol.

[B16] Su TS, Lai CJ, Huang JL, Lin LH, Yauk YK, Chang CM, Lo SJ, Han SH (1989). Hepatitis B virus transcript produced by RNA splicing. J Virol.

[B17] Huang HL, Jeng KS, Hu CP, Tsai CH, Lo SJ, Chang C (2000). Identification and characterization of a structural protein of hepatitis B virus: a polymerase and surface fusion protein encoded by a spliced RNA. Virology.

[B18] Huang ZM, Yen TS (1994). Hepatitis B virus RNA element that facilitates accumulation of surface gene transcripts in the cytoplasm. J Virol.

[B19] Huang ZM, Yen TS (1995). Role of the hepatitis B virus posttranscriptional regulatory element in export of intronless transcripts. Mol Cell Biol.

[B20] Smith GJ, Donello JE, Luck R, Steger G, Hope TJ (1998). The hepatitis B virus post-transcriptional regulatory element contains two conserved RNA stem-loops which are required for function. Nucleic Acids Res.

[B21] Hass M, Hannoun C, Kalinina T, Sommer G, Manegold C, Gunther S (2005). Functional analysis of hepatitis B virus reactivating in hepatitis B surface antigen-negative individuals. Hepatology.

[B22] zu Putlitz J, Tong S, Wands JR (1999). A short region in the genome of hepatitis B virus is critical for maintenance of high transcript levels. Virology.

[B23] Perez I, Lin CH, McAfee JG, Patton JG (1997). Mutation of PTB binding sites causes misregulation of alternative 3' splice site selection in vivo. RNA.

[B24] Abbink TE, Berkhout B (2007). RNA structure modulates splicing efficiency at the HIV-1 major splice donor. J Virol.

[B25] van Hemert FJ, Zaaijer HL, Berkhout B, Lukashov VV (2008). Mosaic amino acid conservation in 3D-structures of surface protein and polymerase of hepatitis B virus. Virology.

[B26] Kortemme T, Kim DE, Baker D (2004). Computational alanine scanning of protein-protein interfaces. Sci STKE.

[B27] Comeau SR, Gatchell DW, Vajda S, Camacho CJ (2004). ClusPro: an automated docking and discrimination method for the prediction of protein complexes. Bioinformatics.

[B28] Zaaijer HL, van Hemert FJ, Koppelman MH, Lukashov VV (2007). Independent evolution of overlapping polymerase and surface protein genes of hepatitis B virus. J Gen Virol.

[B29] Solis AS, Shariat N, Patton JG (2008). Splicing fidelity, enhancers, and disease. Front Biosci.

[B30] Pollicino T, Squadrito G, Cerenzia G, Cacciola I, Raffa G, Craxi A, Farinati F, Missale G, Smedile A, Tiribelli C (2004). Hepatitis B virus maintains its pro-oncogenic properties in the case of occult HBV infection. Gastroenterology.

[B31] Myers R, Clark C, Khan A, Kellam P, Tedder R (2006). Genotyping Hepatitis B virus from whole- and sub-genomic fragments using position-specific scoring matrices in HBV STAR. J Gen Virol.

[B32] Rozanov M, Plikat U, Chappey C, Kochergin A, Tatusova T (2004). A web-based genotyping resource for viral sequences. Nucleic Acids Res.

[B33] Bartholomeusz A, Schaefer S (2004). Hepatitis B virus genotypes: comparison of genotyping methods. Rev Med Virol.

[B34] Thompson JD, Higgins DG, Gibson TJ (1994). CLUSTAL W: improving the sensitivity of progressive multiple sequence alignment through sequence weighting, position-specific gap penalties and weight matrix choice. Nucleic Acids Res.

[B35] Kumar S, Tamura K, Nei M (2004). MEGA3: Integrated software for Molecular Evolutionary Genetics Analysis and sequence alignment. Brief Bioinform.

[B36] Jones DT, Taylor WR, Thornton JM (1992). The rapid generation of mutation data matrices from protein sequences. Comput Appl Biosci.

[B37] Yang Z (2007). PAML 4: phylogenetic analysis by maximum likelihood. Mol Biol Evol.

[B38] Bonneau R, Strauss CE, Rohl CA, Chivian D, Bradley P, Malmstrom L, Robertson T, Baker D (2002). De novo prediction of three-dimensional structures for major protein families. J Mol Biol.

[B39] Chivian D, Baker D (2006). Homology modeling using parametric alignment ensemble generation with consensus and energy-based model selection. Nucleic Acids Res.

[B40] Henrick K, Feng Z, Bluhm WF, Dimitropoulos D, Doreleijers JF, Dutta S, Flippen-Anderson JL, Ionides J, Kamada C, Krissinel E (2008). Remediation of the protein data bank archive. Nucleic Acids Res.

[B41] Wynne SA, Crowther RA, Leslie AG (1999). The crystal structure of the human hepatitis B virus capsid. Mol Cell.

[B42] Brunak S, Engelbrecht J, Knudsen S (1991). Prediction of human mRNA donor and acceptor sites from the DNA sequence. J Mol Biol.

[B43] Hebsgaard SM, Korning PG, Tolstrup N, Engelbrecht J, Rouze P, Brunak S (1996). Splice site prediction in Arabidopsis thaliana pre-mRNA by combining local and global sequence information. Nucleic Acids Res.

[B44] Hall TA (1999). BioEdit: a user-friendly biological sequence alignment editor and analysis program for Windows 95/98/NT. Nucleic Acids Res Symposium Series.

[B45] Zuker M (2003). Mfold web server for nucleic acid folding and hybridization prediction. Nucleic Acids Res.

